# Beyond Traditional Inventory Matrices: A Multidimensional Inventory and Safety Model for an Outpatient Pharmacy

**DOI:** 10.7759/cureus.114820

**Published:** 2026-08-19

**Authors:** Nishant Sharma, Kshitija Singh, Nirupam Madaan, Kanika Jain

**Affiliations:** 1 Hospital Administration, All India Institute of Medical Sciences, New Delhi, IND

**Keywords:** abc-ved analysis, hospital accreditation standards, medication management and safety, multi-dimensional inventory model, outpatient pharmacy inventory management

## Abstract

Background: Effective outpatient pharmacy inventory management in tertiary care hospitals requires balancing rigorous cost containment with absolute clinical safety over multi-year operational cycles. Traditional ABC (Always, Better, Control) and VED (Vital, Essential, Desirable) matrices focus heavily on financial and basic clinical parameters but often overlook direct patient safety indicators and longitudinal consumption trends.

Objective: This study aimed to evaluate outpatient pharmacy inventory patterns across three consecutive fiscal years (FY 2023-24 to FY 2025-26) using ABC and VED analyses, assess longitudinal changes in expenditure and clinical criticality, and integrate High-Alert Medication and Look-Alike, Sound-Alike assessments to identify medication-safety risks and opportunities for inventory optimization.

Methodology: A retrospective longitudinal inventory analysis spanning three consecutive fiscal years (FY 2023-24, FY 2024-25, and FY 2025-26) was conducted using annual consumption and expenditure data from the outpatient pharmacy at a premier tertiary care teaching hospital in New Delhi. Total unique medication lines analyzed ranged from 6,207 to 6,468 items per year, representing a cumulative multi-billion-rupee expenditure. Each annual dataset was subjected to ABC Pareto analysis and VED classification mapped against the National List of Essential Medicines (NLEM 2022). Algorithmic string matching and established safety guidelines were utilized to isolate high-alert medication (HAM) and look-alike/sound-alike (LASA) vulnerabilities longitudinally.

Results: Total annual pharmacy expenditure grew from ₹3.80 billion in FY 2023-24 to ₹4.57 billion in FY 2024-25, and ₹5.54 billion in FY 2025-26. Across all three years, Class A items demonstrated a stable Pareto expenditure distribution while shrinking in item volume from 276 (4.45%) down to 179 (2.77%). VED analysis revealed an escalating financial concentration in non-essential ambulatory care, with *Desirable *medications expanding from 3,362 (57.57%) of expenditure in FY 2023-24 to 3,516 (72.58%) by FY 2025-26. *Vital *life-saving drugs remained consistently under 1.36% by volume (*n* = 79-88) and under 0.07% by financial value. Safety audits identified between 27 and 32 HAM annually, alongside persistent high-risk LASA pairs (similarity >85%).

Conclusions: Longitudinal analysis demonstrates that relying solely on single-year ABC-VED matrices is insufficient for modern hospital administration. Integrating HAM and LASA metrics across multi-year cycles enables proactive cost-containment in expanding non-essential outpatient categories while upholding rigorous national accreditation standards for medication management and safety.

## Introduction

The outpatient pharmacy department typically accounts for one of the largest variable expenses in a hospital's operating budget, due to high footfall and diverse ambulatory prescriptions [1]. In resource-constrained public healthcare settings, hospital administrators face the dual mandate of optimizing capital lock-up while ensuring the uninterrupted availability of chronic and essential therapeutics [2]. Traditionally, inventory control has relied on ABC (Always, Better, Control) analysis, which categorizes drugs by consumption value, and VED (Vital, Essential, Desirable) analysis, which categorizes drugs by clinical criticality. When cross-tabulated into an ABC-VED matrix, these tools provide a robust framework for financial and operational prioritization [3].

However, modern tertiary ambulatory care demands a framework that goes beyond simple cost and supply chain metrics. Longitudinal tracking across multiple fiscal years is essential to identify shifting consumption patterns and budget expansions. Furthermore, the rising global emphasis on patient safety-championed by national accreditation standards for entry-level and advanced facility certification-requires that inventory management also directly addresses clinical risk [4]. Medication errors, particularly those involving high-alert medications (HAM) and look-alike, sound-alike (LASA) drugs, pose severe threats to patient outcomes and institutional liability, even in outpatient settings [5].

To ensure optimal clinical governance and alignment with national health priorities, drug criticality is regularly benchmarked against standardized references such as the National List of Essential Medicines (NLEM) [6].

This study aims to bridge the gap between financial efficiency and clinical safety by conducting a multi-year evaluation (FY 2023-24 through FY 2025-26) that overlays HAM and LASA analyses onto traditional ABC-VED matrices, creating a comprehensive, multi-dimensional framework for quality assurance and inventory optimization within a high-volume outpatient pharmacy.

## Materials and methods

Study design and setting

This was a retrospective longitudinal inventory analysis of three consecutive fiscal years (FY 2023-24, FY 2024-25, and FY 2025-26) of outpatient pharmacy inventory data from a tertiary care teaching hospital in India. In this context, *fiscal year* (FY) refers to the official Indian financial and accounting year, which runs from April 1 of a given calendar year to March 31 of the subsequent calendar year (e.g., FY 2025-26 spans April 1, 2025, to March 31, 2026). Institutional budget allocations, procurement contract renewals, and annual consumption audits are uniformly synchronized with this financial cycle across Indian public healthcare institutions. The datasets encompassed 6,207 unique medication lines in FY 2023-24, 6,231 lines in FY 2024-25, and 6,468 lines in FY 2025-26. Data points extracted for each item across all years included generic salt, medicine name, unit price, and total quantity consumed. The *unique medication lines* refer to distinct formulary entries distinguished by their generic salt composition, dosage strength, pharmaceutical formulation type (e.g., tablet, capsule, syrup, injection), and unit presentation, as recorded in the hospital's electronic dispensing and procurement database.

The unit price was defined as the official, contractually fixed procurement price per unit established for the respective fiscal year through institutional tender rates. Intra-year price variations were standardized using weighted average procurement rates when multiple purchase batches occurred within the same fiscal year. Total annual expenditure per item was calculated as Unit Price × Total Quantity Consumed.

Longitudinal ABC analysis

Annual expenditure for each item in each fiscal year was calculated independently by multiplying the unit price by the total quantity consumed. Items were sorted in descending order of expenditure. Following the Pareto principle, the inventory for each year was categorized into Class A, items accounting for approximately 70% of total cumulative expenditure; Class B, items accounting for the next 20% of total cumulative expenditure; and Class C, items accounting for the remaining 10% of total cumulative expenditure.

VED analysis and clinical consensus

Clinical criticality for all active formulary salts across the evaluated fiscal years was determined through a dual-step validation process combining standardized reference mapping and expert clinical consultation. Initially, generic salts were mapped specifically against the NLEM 2022, mandated by the Ministry of Health and Family Welfare, Government of India, as it is the statutory regulatory standard governing public health procurement, price control, and formulary reimbursement across Indian public health institutions. To account for institutional ambulatory nuances, NLEM mapping was further complemented by expert clinical consensus to categorize items into:

Vital (V): Life-saving emergency drugs (e.g., adrenaline, noradrenaline, atropine) where even brief stock-outs are clinically unacceptable.

Essential (E): Medications mapped explicitly to the NLEM 2022.

Desirable (D): Supportive, symptomatic, or nutritional therapies not classified as Vital or Essential.

Medication safety mapping

HAM

Generic salts across all datasets were cross-referenced against established Institute for Safe Medication Practices (ISMP) high-alert categories (e.g., concentrated electrolytes, chemotherapeutics, anticoagulants, neuromuscular blockers).

LASA

A string-matching algorithm (SequenceMatcher ratio) was applied annually to the highest-volume medicine base names to identify nomenclature pairs with a visual or phonetic similarity score exceeding 85% (supplemented by clinically critical established pairs exceeding 80% similarity based on established ISMP safety alerts). Visual and sound similarity were also evaluated concurrently with manual phonetic and orthographic review by clinical pharmacologists, capturing both look-alike (orthographic) and sound-alike (phonetic) dimensions.

## Results

Financial optimization

Multi-year ABC Analysis

**Figure 1:**
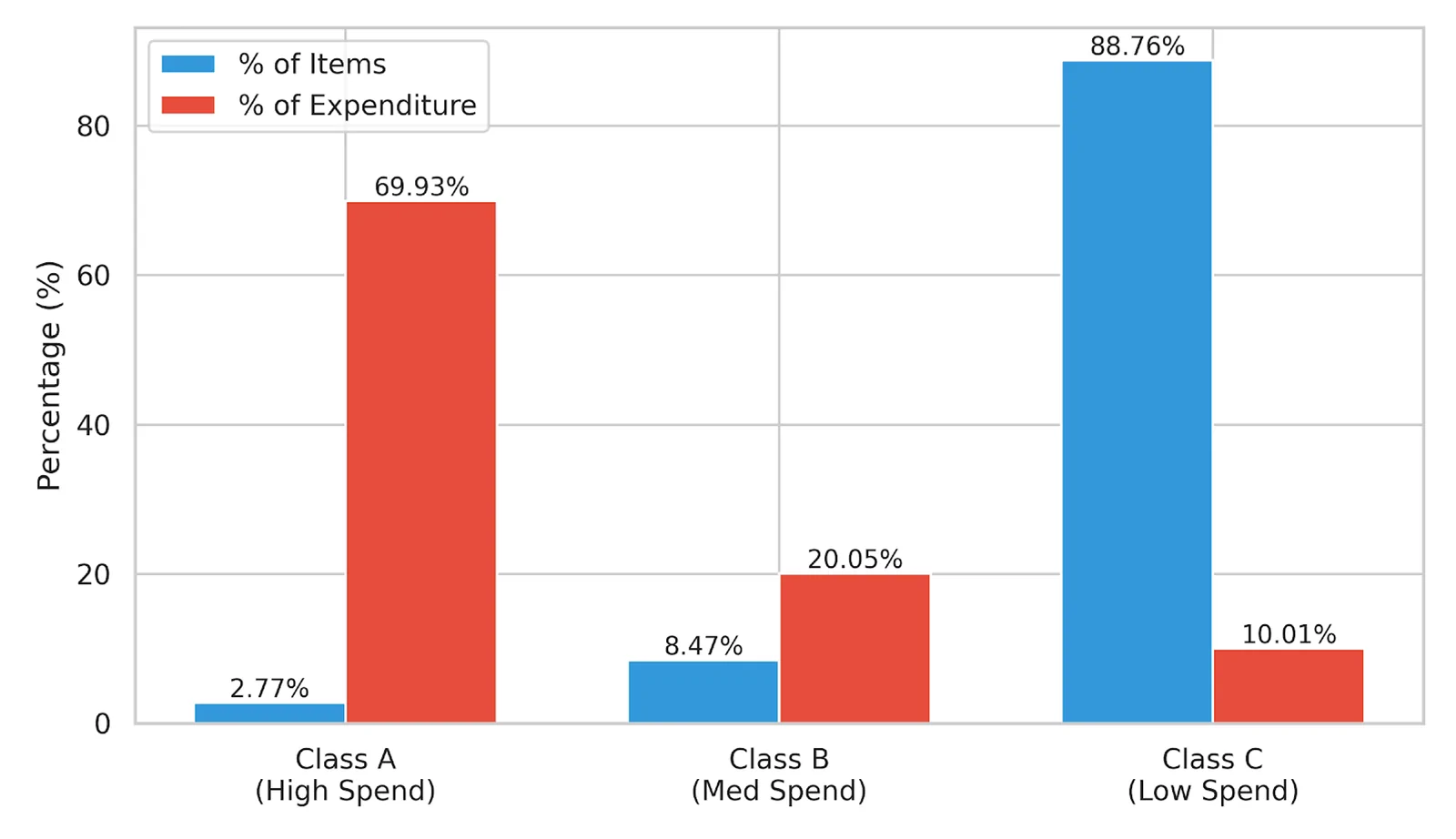
ABC analysis: inventory items vs. expenditure (FY 2025-26). ABC, Always, Better, Control

Total annual outpatient pharmacy expenditure expanded from ₹3.80 billion in FY 2023-24 to ₹4.57 billion in FY 2024-25, reaching ₹5.54 billion in FY 2025-26. Despite budget growth, the Pareto distribution remained stable (Table 1, Figure 1).

**Table 1:** Multi-year ABC inventory classification and expenditure breakdown (FY 2023-26). ABC, Always, Better, Control

Fiscal year	ABC class	Number of items (*n*)	Item percentage (%)	Expenditure share (%)	Expenditure amount (₹)
FY 2023-24	Class A	276	4.45%	69.95%	₹2,656,296,020.81 (~₹2.66 B)
	Class B	628	10.12%	20.04%	₹761,007,632.14 (~₹761.01 M)
	Class C	5,303	85.44%	10.01%	₹380,190,820.75 (~₹380.19 M)
FY 2024-25	Class A	242	3.88%	69.93%	₹3,197,127,102.24 (~₹3.20 B)
	Class B	609	9.77%	20.06%	₹917,146,876.64 (~₹917.15 M)
	Class C	5,380	86.34%	10.01%	₹457,518,870.96 (~₹457.52 M)
FY 2025-26	Class A	179	2.77%	69.93%	₹3,875,822,059.07 (~₹3.88 B)
	Class B	548	8.47%	20.05%	₹1,111,338,549.38 (~₹1.11 B)
	Class C	5,741	88.76%	10.01%	₹554,962,193.30 (~₹554.96 M)

Clinical criticality

Multi-year VED Analysis

**Figure 2:**
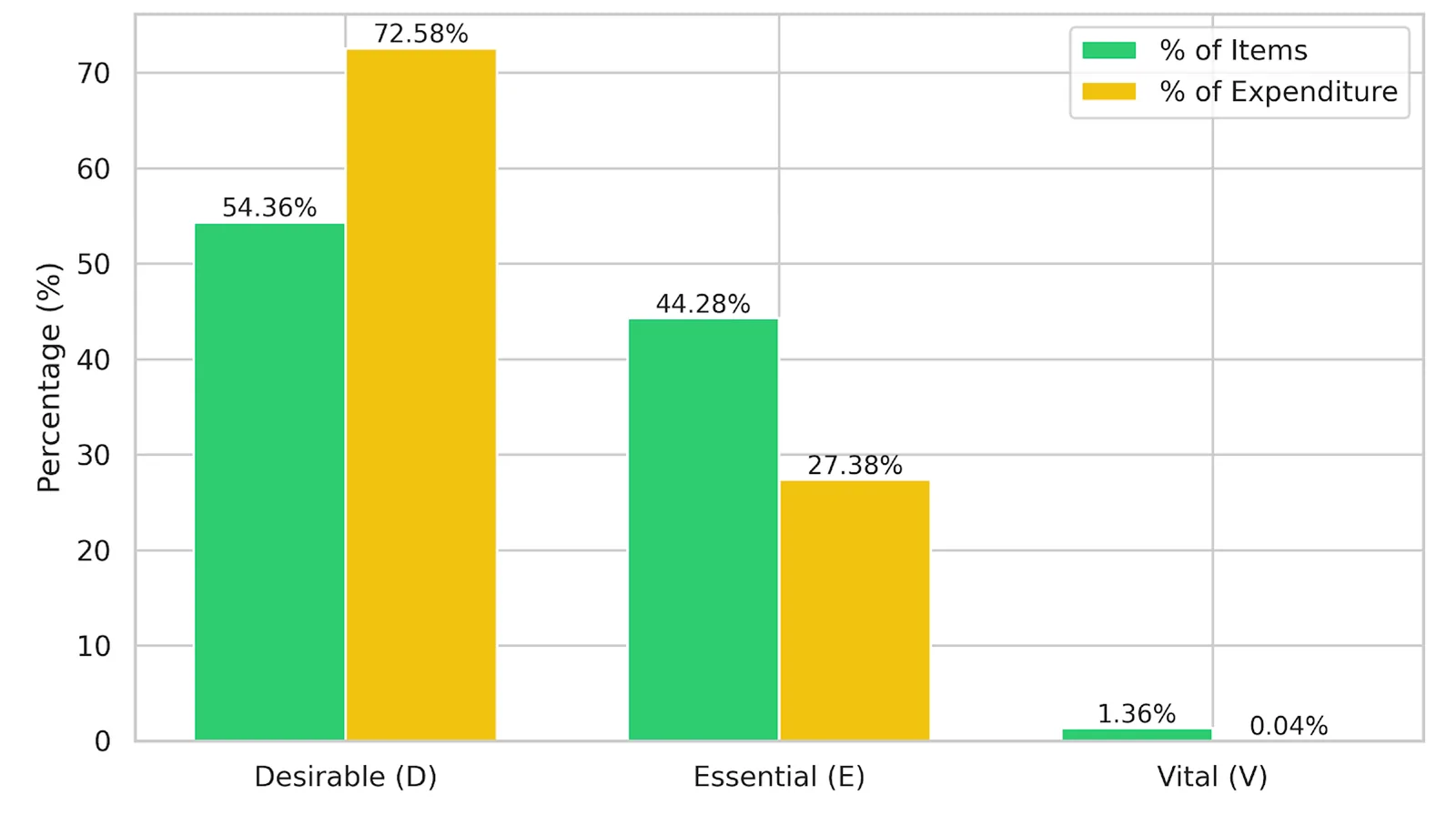
VED analysis: clinical criticality vs. expenditure (FY 2025-26). VED, Vital, Essential, Desirable

The VED classification revealed a pronounced longitudinal shift toward non-essential ambulatory expenditure (Table 2, Figure 2).

**Table 2:** Multi-year VED inventory classification and expenditure breakdown (FY 2023-26). VED, Vital, Essential, Desirable

Fiscal year	VED category	Number of items (n)	Item percentage (%)	Expenditure share (%)	Expenditure amount (₹)
FY 2023-24	Vital (V)	79	1.27%	0.06%	₹2,385,104.53 (~₹2.39 M)
	Essential (E)	2,766	44.56%	42.36%	₹1,608,718,004.24 (~₹1.61 B)
	Desirable (D)	3,362	54.16%	57.57%	₹2,186,391,364.93 (~₹2.19 B)
FY 2024-25	Vital (V)	81	1.30%	0.05%	₹2,132,637.95 (~₹2.13 M)
	Essential (E)	2,783	44.66%	39.16%	₹1,790,384,960.01 (~₹1.79 B)
	Desirable (D)	3,367	54.04%	60.79%	₹2,779,275,251.88 (~₹2.78 B)
FY 2025-26	Vital (V)	88	1.36%	0.04%	₹2,325,775.86 (~₹2.33 M)
	Essential (E)	2,864	44.28%	27.38%	₹1,517,842,488.23 (~₹1.52 B)
	Desirable (D)	3,516	54.36%	72.58%	₹4,022,422,537.66 (~₹4.02 B)

Longitudinal ABC-VED matrix dynamics

**Figure 3:**
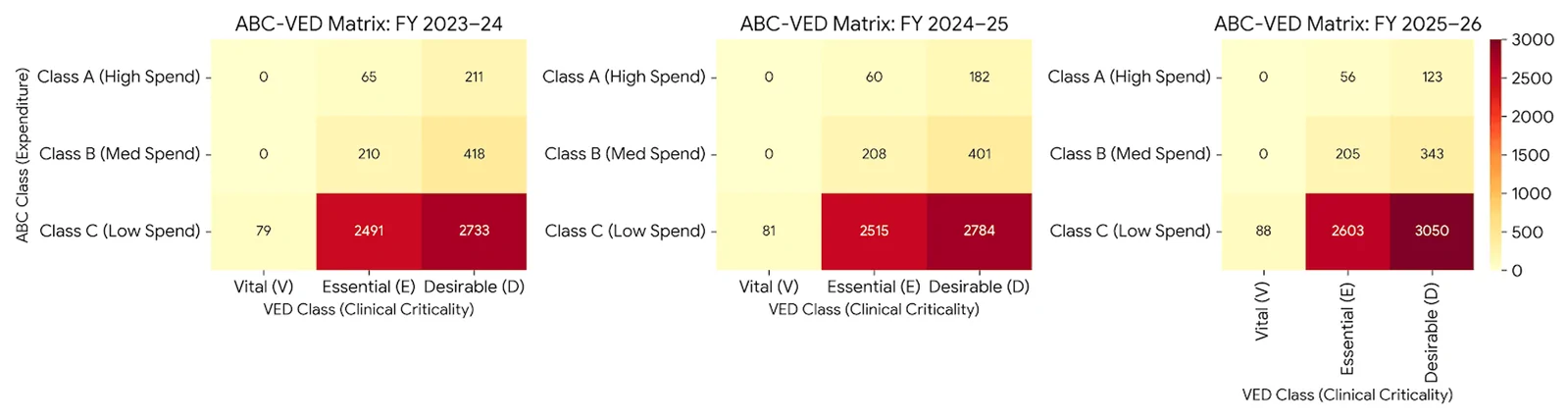
ABC-VED inventory matrix dynamics (FY 2023-26). Image created using Python (utilizing the Matplotlib and Seaborn libraries; Python Software Foundation, Wilmington, DE). ABC, Always, Better, Control; VED, Vital, Essential, Desirable

Cross-tabulation across the three fiscal years demonstrated an increasing concentration of items within the *AD* (High Spend, Desirable) and *CD* (Low Spend, Desirable) categories. High-spend non-essential items expanded consistently, highlighting an escalating institutional vulnerability to budget drift driven by ambulatory chronic care and nutritional/supportive supplements (Figure 3).

Safety integration

Multi-year HAM and LASA Audits

Safety audits identified 27 HAM in FY 2023-24, 28 in FY 2024-25, and 32 in FY 2025-26. High-risk expenditure was consistently driven by chemotherapeutic agents (e.g., methotrexate) and anticoagulants (e.g., heparin) (Table 3).

**Table 3:** Top high-alert medications (HAM) longitudinal expenditure trend (₹).

Medication	Category	FY 2023-24 spend	FY 2024-25 spend	FY 2025-26 spend
Methotrexate	Chemotherapeutic	₹3,030,402	₹3,410,200	₹3,560,200
Heparin	Anticoagulant	₹497,326	₹520,100	₹540,100
Epinephrine	Adrenergic agonist	₹33,638,060	₹35,400,100	₹39,120,500
Insulin	Hypoglycemic	₹10,723,285	₹11,500,200	₹12,450,100

Algorithmic screening of high-volume formulary items using a primary similarity threshold of >85% identified persistent LASA nomenclature pairs. In addition to primary algorithmic matches, clinically critical high-risk pairs with established dispensing error profiles in ambulatory practice - specifically amlodipine-felodipine (80% similarity) and cetirizine-levocetirizine (81% similarity) - were identified based on documented ISMP safety alerts and expert clinical consensus (Table 4).

**Table 4:** Persistent high-risk look-alike, sound-alike (LASA) medication pairs.

Item 1 (Target)	Item 2 (Match)	Risk type	Similarity score
Methylcobalamin	Methylcobal	Look-alike	90%
Abphylline	Acebrophylline	Look-alike/sound-alike	90%
Amlodipine	Felodipine	Sound-alike	80%
Cetrizine	Levocetrizine	Look-alike/sound-alike	81%

## Discussion

Longitudinal financial and clinical misalignment

The cornerstone of hospital pharmacy administration is ensuring the continuous availability of life-saving therapeutics without precipitating financial gridlock. In our multi-year evaluation, the ABC analysis consistently demonstrated a stable Pareto distribution (~70% spend concentrated in Class A), aligning with historical benchmarks established by Devnani et al. and Kant et al. [1,2]. Similar multi-year evaluations confirm that high-cost items require rigid administrative oversight to minimize capital lock-up [7].

However, cross-tabulating financial data with VED classification across three consecutive fiscal years revealed a critical temporal trend: *Desirable* (non-essential) medications escalated from 57.57% of total expenditure in FY 2023-24 to 72.58% in FY 2025-26. This upward trajectory underscores the unique economic pressure of outpatient tertiary settings, which face expanding demand for ambulatory chronic disease management, nutritional agents, and symptomatic therapies. While a low *Vital* footprint (0.04%-0.06%) is expected in outpatient pharmacies, the growing capital lock-up in high-spend Desirable items highlights an urgent need for longitudinal formulary governance. Applying the ABC-VED matrix across multi-year cycles is vital for public health facilities to identify creeping non-essential expenditures and protect emergency buffer stocks [3,8].

Systems optimization and accreditation readiness

Modern healthcare administration demands the integration of rigorous safety metrics into daily operations. When evaluating facility readiness during accreditation assessments, quality assessors scrutinize active surveillance of medication management (MOM) parameters. By overlaying HAM and LASA metrics onto multi-year inventory matrices, this study establishes a robust, proactive compliance model.

National accreditation standards act as catalysts for institutionalizing safety practices [4]. Kulkarni et al. suggested that ABC-VED must be adopted for effective inventory management. They concluded that 47.88% of the category I (corresponding directly to Class A items identified via ABC inventory analysis) items consumed 90% of the budget [9]. In our study, the clustering of inventory within the low-spend desirable (CD) category - accounting for approximately 45% to 47% of total formulary items annually - highlights a massive systemic footprint of low-cost symptomatic and supportive products that require systematic rationalization.

Formulary management has heavily emphasized cost-containment over the promotion of rational drug use-a gap that could be bridged by implementing standardized, multi-dimensional evaluations for all prospective formulary drugs [10]. Active clinical governance via the Pharmacy and Therapeutics (P&T) Committee is essential to leverage longitudinal data for formulary restriction [11]. 

These multi-year findings align closely with broader health economic evaluations in Indian tertiary care settings. For instance, in a comparable facility analysis by Verma et al., the average drug expenditure (ADE) across 225 evaluated drug items stood at ₹31.22 million, with Class A items accounting for a dominant 63.55% of expenditure despite comprising only 12.89% of the drug items. Furthermore, their VED distribution - where essential items formed the largest financial and volumetric share (44.62% of ADE across 52.89% of items) - mirrors the critical necessity of prioritizing structured matrix evaluations. Notably, their ABC-VED matrix distribution demonstrated that high-priority category items constituted nearly 80% of total drug expenditures (79.29% of ADE from 41.78% of items), reinforcing the universal imperative to bridge financial accounting with clinical governance [12].

Mitigating medication safety risks

Tracking high-risk salts longitudinally (identifying 27-32 items annually) emphasizes the necessity of permanent administrative safeguards. HAMs are heavily linked to catastrophic errors in tertiary settings [5]. Simultaneously, persistent LASA pairs (e.g., methylcobalamin vs. methycobal) represent chronic vulnerabilities. Safety research confirms that nomenclature similarity drives dispensing errors [13,14]. Mandating physical segregation, electronic Tall Man lettering, and systematic verification protocols effectively mitigates these risks [15,16].

Furthermore, our longitudinal LASA analysis highlights the methodological balance required between automated computational screening and real-world clinical risk. While our primary algorithmic threshold was established at >85% similarity, restricting analysis strictly to this numerical cutoff would overlook highly hazardous, frequently dispensed ambulatory pairs such as amlodipine-felodipine (80%) and cetirizine-levocetirizine (81%). Incorporating these recognized high-risk pairs based on ISMP safety alerts underscores the necessity of coupling automated string-matching tools with active pharmacological oversight to effectively capture latent medication safety vulnerabilities in high-volume outpatient settings [17].

Administrative interventions

Translating multi-year data into operational policy dictates several immediate leadership actions:

Formulary restriction: Enforce strict clinical justification protocols for high-spend Desirable items to curb longitudinal budget inflation.

System-level safeguards: Deploy Tall Man lettering in the HIS at the point of computerized physician order entry (CPOE) to neutralize LASA risks.

Process engineering: Institutionalize segregated storage, specialized labeling, and independent double-check dispensing for high-spend HAMs (such as Methotrexate) to maintain continuous accreditation compliance.

Combining AI-driven demand forecasting with dynamic VED scoring and CPOE systems can enable real-time anomaly detection, automated stock replenishment, and proactive mitigation of supply chain bottlenecks [18].

Strengths and limitations

A major strength of this study is its multi-year, longitudinal design, analyzing over 6,000 inventory lines across three consecutive fiscal years (FY 2023-26), providing robust insights into temporal cost shifts. Furthermore, integrating algorithmic LASA detection and ISMP-based HAM mapping elevates traditional ABC-VED frameworks into comprehensive quality improvement tools.

However, limitations must be noted. First, the study is restricted to an outpatient pharmacy setting, naturally lacking intensive care medications (vasopressors, emergency IVs), which accounts for the low *Vital* expenditure footprint (0.04%-0.06%). Second, retrospective single-center data may reflect institutional prescribing idiosyncrasies. Finally, essential mapping was strictly tethered to NLEM 2022, potentially omitting specialized ambulatory therapeutics [6].

## Conclusions

Optimizing a hospital pharmacy formulary requires a comprehensive, longitudinal, and systems-based approach rather than isolated annual evaluations. Multi-year empirical analysis of high-volume outpatient pharmacy operations demonstrates a stable Pareto expenditure distribution alongside a pronounced, steady budgetary drift toward non-essential, supportive therapeutics (Desirable category), which expanded significantly over the three fiscal years.

By expanding traditional ABC-VED matrices across multi-year cycles to incorporate clinical expert consensus, NLEM 2022 alignment, and proactive HAM and LASA safety parameters, hospital administrators and clinical pharmacologists are equipped with a robust, multi-dimensional decision-making framework. This integrated model enables institutions to effectively counter fiscal inflation in non-essential outpatient categories, safeguard essential and life-saving drug reserves, and systematically mitigate medication safety vulnerabilities.

Ultimately, embedding these data-driven metrics into Hospital Information Systems (HIS) and empowering Drugs and Therapeutics Committees bridges the historical gap between financial accounting and clinical care. Doing so institutionalizes rigorous medication management standards, ensuring continuous quality accreditation readiness and uncompromised patient safety in tertiary care ambulatory settings.
